# Withaferin-A Treatment Alleviates TAR DNA-Binding Protein-43 Pathology and Improves Cognitive Function in a Mouse Model of FTLD

**DOI:** 10.1007/s13311-020-00952-0

**Published:** 2020-10-19

**Authors:** Sunny Kumar, Daniel Phaneuf, Jean-Pierre Julien

**Affiliations:** 1 CERVO Brain Research Centre, Laval University, Quebec City, QC Canada; 2 Department of Psychiatry and Neuroscience, Canada Research Chair in Neurodegeneration, Université Laval, 2601, Chemin de la Canardière, Québec City, Québec G1J 2G3 Canada

**Keywords:** Amyotrophic lateral sclerosis, frontotemporal dementia, TDP-43, withaferin-A, *Withania somnifera*, NF-κB, autophagy

## Abstract

Withaferin-A, an active withanolide derived from the medicinal herbal plant *Withania somnifera* induces autophagy, reduces TDP-43 proteinopathy, and improves cognitive function in transgenic mice expressing mutant TDP-43 modelling FTLD. TDP-43 is a nuclear DNA/RNA-binding protein with cellular functions in RNA transcription and splicing. Abnormal cytoplasmic aggregates of TDP-43 occur in several neurodegenerative diseases including amyotrophic lateral sclerosis (ALS), frontotemporal lobar degeneration (FTLD), and limbic-predominant age-related TDP-43 encephalopathy (LATE). To date, no effective treatment is available for TDP-43 proteinopathies. Here, we tested the effects of withaferin-A (WFA), an active withanolide extracted from the medicinal herbal plant *Withania somnifera*, in a transgenic mouse model of FTLD expressing a genomic fragment encoding mutant TDP-43^G348C^. WFA treatment ameliorated the cognitive performance of the TDP-43^G348C^ mice, and it reduced NF-κB activity and neuroinflammation in the brain. WFA alleviated TDP-43 pathology while it boosted the levels of the autophagic marker LC3BII in the brain. These data suggest that WFA and perhaps other autophagy inducers should be considered as potential therapy for neurodegenerative diseases with TDP-43 pathology.

**Electronic supplementary material:**

The online version of this article (10.1007/s13311-020-00952-0) contains supplementary material, which is available to authorized users.

## Introduction

TDP-43 is a DNA/RNA-binding protein localized to the nuclear compartment of the cells. TDP-43 functionally helps in the regulation of RNA transcription, splicing, and trafficking [1]. Ubiquitin-positive inclusions of TDP-43 in the cytoplasm are a hallmark of several neurodegenerative diseases including ALS, FTLD, and LATE [2, 3]. Approximately 50% of FTLD and 90 to 95% of ALS patients exhibit TDP-43 proteinopathy [4, 5]. So far, the drugs available for ALS are riluzole and edaravone [6, 7] but they extend the lifespan of patients by only a few months [6].

Many studies have shown that boosting the clearance of TDP-43 aggregates alleviated the pathology in mice models of ALS/FTLD [8–10]. TDP-43 protein aggregates in the cells are targets of both autophagy and proteasome-dependent degradation [10–12]. Autophagy is an intracellular phenomenon, which contributes to the removal of cellular proteins and organelles by processing the formation of autophagosomes followed by lysosome-mediated degradation [13]. A recent study has shown that TDP-43 functional loss leads to suppression of the autophagic flux [14]. Induction of autophagic markers has been shown to reduce TDP-43 pathology and to improve the cognitive function in mouse models of ALS/FTLD [8, 9].

Alternatively, targeting the NF-κB pathway has also shown beneficial effects in mouse models of ALS/FTLD [9, 15–17]. Treatment with root extract of the medicinal herbal plant *Withania somnifera* reduced NF-κB activity and alleviated TDP-43 proteinopathy while it ameliorated motor performance in a transgenic mouse model of ALS/FTLD with TDP-43 pathology [16]. In addition to ALS, *Withania somnifera* has shown therapeutic potential in many different neurodegenerative diseases including Alzheimer’s disease [16, 18]. Furthermore, withaferin-A (WFA), an active steroid lactone derived from the *Withania somnifera*, also conferred therapeutic benefits in different mice models of ALS [15, 19]. Previously, our group reported that WFA treatment reduced inflammation in the spinal cord and rescued the motor pathology in transgenic mice expressing TDP-43 mutants, models of ALS/FTLD [15]. A recent study has shown that WFA treatment protected dopaminergic neurons and motor function in aging rats [20]. Considering the reported neuroprotective effect of WFA, we tested the effects of WFA treatment on TDP-43 proteinopathy, autophagy, and NF-κB in mice expressing TDP-43^G348C^. WFA treatment of TDP-43^G348C^ mice caused a reduction of TDP-43 cytoplasmic mislocalization and aggregation in the brain and improved memory function. WFA boosted the levels of autophagic marker LC3BII in the brain while it reduced NF-κB-dependent neuroinflammation.

## Methods

### Drug and Cell Lines

Withaferin-A (WFA) was obtained from Sigma–Aldrich (Saint Louis, USA). The purity of WTA is > 94%. BV2/NF-κB-Luc and NSC-34/NF-κB-Luc stable cell lines were generated in the lab using pGL4.32[luc2P/NF-κB–RE/Hygro] plasmid DNA (Promega, Madison, WI, USA). The vector expressed 5 copies of the NF-ĸB response element that drives transcription of the luciferase reporter gene luc2P. All the cell lines used were from the American Type Culture Collection (ATCC).

### Cell Culture and Treatment

BV2/NF-κB-Luc and NSC-34/NF-κB-Luc cells were cultured in Gibco high-glucose DMEM (Life Technologies) with 10% FBS. Culture media for all cell lines were supplemented with 1% penicillin/streptomycin and 1% sodium pyruvate (Gibco). For the clonal selection of BV2/NF-κB-Luc and NSC-34/NF-κB-Luc cells, media were supplemented with 0.1 mg/mL hygromycin-B. WFA was dissolved in dimethyl sulfoxide (DMSO) solution. The final amount of DMSO in the media was equivalent in drug- and vehicle-treated samples. All the treatment was conducted in serum-free media.

### NF-κB Signaling and MTS Assay

For NF-κB-luciferase reporter assays, BV2/NF-κB-Luc (5 × 10^4^ cells per well) and NSC-34/NF-κB-Luc (5 × 10^4^ cells per well) cells were seeded in 24-well plates. We tested different concentrations of WFA to define a maximum dose that was nontoxic for cultured cells using a MTS cell viability assay as described below. For the BV2 cells, it was a dose of 2.5 μM, and for NSC-34 cells, it was 1.0 μM. The day after seeding, BV2/NF-κB-Luc cells were pretreated with DMSO or LPS (500 ng/ml) for 2 h and then treated with WFA for an additional 2 h. At the end, luciferase activity was measured using the One-Glo Luciferase Assay (Promega, Madison, WI) according to the manufacturer’s instructions.

Twenty-four hours later being plated, NSC-34/NF-κB-Luc cells were pretreated with DMSO or WFA (1 μM) for 20 min and then treated with TNF-α (40 ng/ml) for 4 h. At the end, luciferase activity was measured using the One-Glo Luciferase Assay (Promega, Madison, WI) according to the manufacturer’s instructions.

For the MTS assay, we followed the same treatment paradigm shown above with both BV2/NF-κB-Luc and NSC-34/NF-κB-Luc cells. At the end of treatment for the MTS assay, we used [3-(4,5-dimethylthiazol-2-yl)-5-(3carboxymethoxyphenyl)-2-(4-sulfophenyl)-2H-tetrazolium], as per the manufacturer’s instructions (Promega). The absorbance of the formazan adduct formed was determined at 490 nm using an EnSpire 2300 Multilabel reader (Perkin Elmer, Waltham, MA, USA). Values were expressed as the percentage relative to controls.

### Mouse Treatment

The TDP-43^G348C^ mice were used for this study. In this model, TDP-43 proteinopathy starts around the age of 8 to 10 months followed by cognitive impairment at the age of 12 months [21]. Our study utilized 16 mice, which were randomly divided into the experiment group and the vehicle group, 8 in each. An equal number of male and female mice were used for the experiment in each treatment group (4 males and 4 females). The safety profile of WFA in animals has already been proven and widely accepted. For testing the therapeutic potential, WFA was administrated to the experiment group (*n* = 8) intraperitoneally 5 mg/kg of body weight once every 2 days for 8 weeks. On the other hand, the vehicle group (*n* = 8) was treated with saline. The Animal Care Ethics Committee of Université Laval approved all in vivo experimental protocols. Experiments were carried out in accordance with the Guide for the Care and Use of Experimental Animals of the Canadian Council on Animal Care.

### Passive Avoidance Test

Initially, the mice were conditioned in a light–dark chamber. The next day, the mice were conditioned with foot electric shock when they enter the dark chamber. At the end, one-trial passive avoidance was performed as described earlier [16, 22] to estimate the latency (in seconds) of mice entering the dark chamber.

After WFA treatment and behavioral tests, a total of 8 animals were processed for tissue collection either for immunofluorescence microscopy or western blotting. At this step, we blindly split each group into two subgroups. We processed 4 mice from each group for western blotting and the other 4 mice from each group for immunofluorescence microscopy. For microscopy, trans-cardial perfusion was performed with 4% PFA. Further, 3 mice (2 females and 1 male) were selected from each group for the microscopy analysis. For autophagy analysis, *n* = 3 to 4 were used from the tissue processed for western blotting.

### Western Blot Assay

To check protein levels, the brain tissue from each group were lysed in RIPA lysis buffer containing protease and phosphatase inhibitor cocktail. To compare the level of aggregation of insoluble TDP-43 in different groups, RIPA-insoluble/soluble fractionation was performed. Further, the insoluble fraction was solubilized in 6 M urea buffer. For performing the SDS-PAGE protein concentration was evaluated using Bradford protein assay. The SDS-PAGE was performed using 20 to 25 μg total protein extract from each sample. Further, the separated protein extract was transferred to polyvinylidene fluoride (PVDF) membranes. Once the proteins were transferred, the PVDF membrane was blocked using 5% BSA in PBST (0.1% Tween 20). Corresponding primary antibodies (1:1000) were used to probe mouse monoclonal hTDP-43 (Abnova, Taiwan), LC3 (Novus Biologicals, USA), Beclin-1 (Novus Biologicals, USA), p62 (Millipore, USA), ATG-5 (Millipore, USA), and Actin (Millipore, USA). Subsequently, HRP-conjugated anti-rabbit antibody or HRP anti-mouse antibody was used for the following incubation. The blots were developed with ECL detection reagents and visualized with a StarBright Blue 520 (Bio-Rad Laboratories, USA). All band intensities were quantified using the ImageJ lab software.

### Immunofluorescence Staining

Thirty-micrometer-thick sagittal cryosections of mouse brain were cut and mounted on glass slides. The sections were pretreated with 0.01 mol/L citrate buffer (pH = 6.0) for 20 min and then were blocked for 1 h with 10% goat serum in PBST (0.25% triton-X100). Following blocking, the sections were incubated with primary antibodies, overnight at 4 °C. The next day, the sections were washed and incubated with Alexa-Fluor fluorescent dye-conjugated secondary antibodies Invitrogen, USA; 1:500) in PBS for 90 min at room temperature. The primary antibodies were specific to mouse monoclonal human TDP-43 (Abnova, Taiwan; 1:500), mouse polyclonal GFAP (Cell Signaling Technologies, USA; 1:500), rabbit polyclonal Iba1 (Wako Chemicals, USA; 1:500), mouse monoclonal NeuN (Cell Signaling Technologies, USA; 1:1000) Nuclear factor kappa-B (NF-κB) (Santa Cruz;1:1000), phospho-NF-κB (Ser 536) (Cell Signaling Technologies, USA; 1:500), arginase-1 (Santa Cruz; 1:1000), and Ym-1 (Stem Cell Technologies, Canada;1:500) protein markers. Four equidistant sections for the hippocampus and six equidistant sections of the cortex were assessed for every mouse. All sections were imaged using confocal microscopy (LSM microscope 700, Zeiss). The ImageJ software was used for analyzing and quantifying the immunoreactive areas.

### Statistical Analysis

For all the statistical analyses, Prism 5.0 software (GraphPad, La Jolla, CA, USA) was used. For comparing 2 groups, the unpaired two-tailed *t* test was used, while for comparing multiple groups, one-way analysis of variance with Bonferroni’s or Tukey’s post test was used. A minimum of up to 0.05 *p* value was statistically significant.

## Results

### WFA Treatment Inhibits NF-κB Activation in both Microglial and Cultured Neuronal Cells

**Fig. 1:**
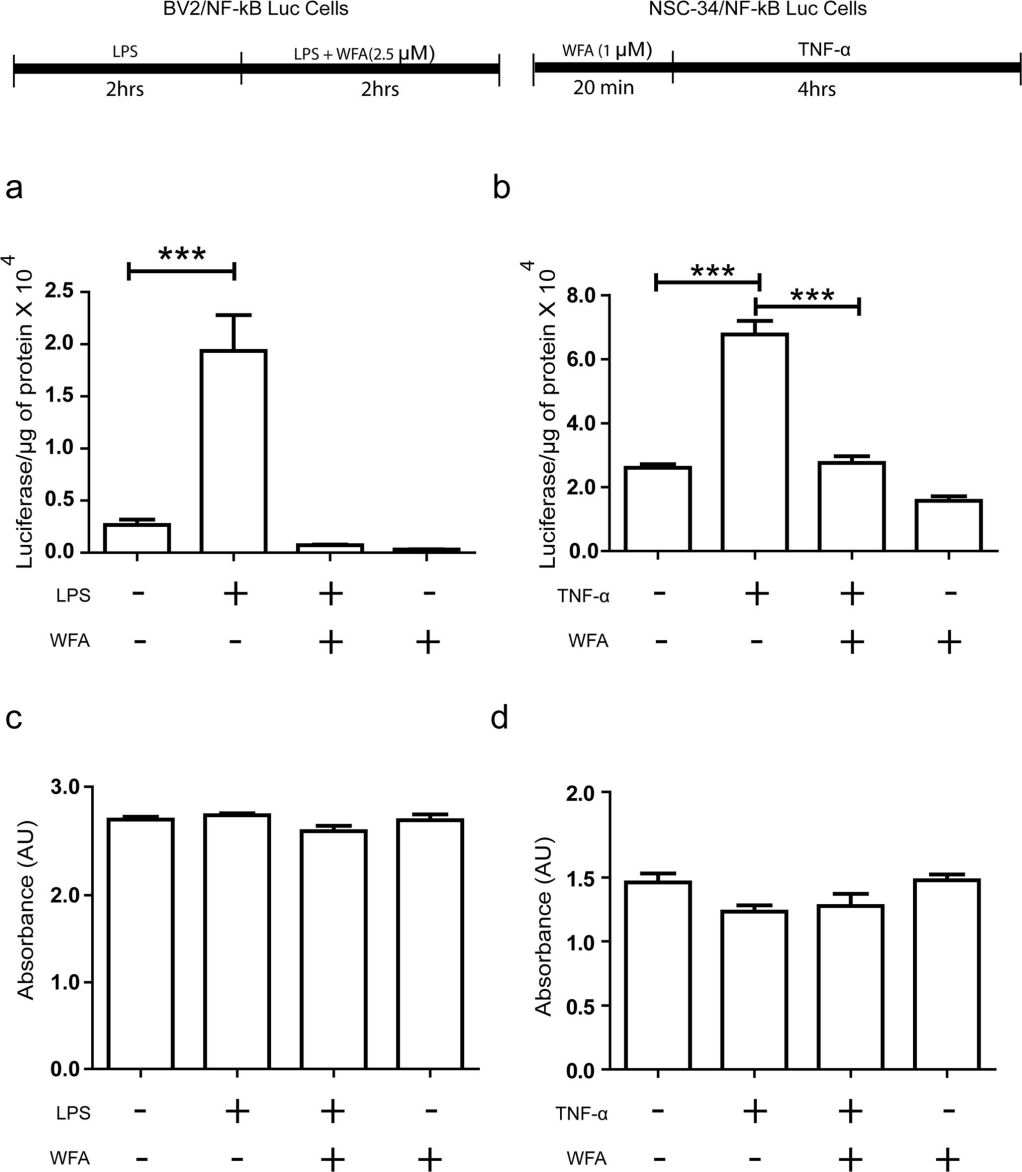
WFA treatment Inhibits NF-κB Activation in both microglial and neuronal cultured cells. (a) BV2/NF-κB luc cells was pretreated with LPS bacterial LPS (500 ng/ml) for 2 h. Posttreatment of LPS stimulated cells were treated with WFA in LPS containing media for 2 h (*n* = 4; one way ANOVA; Tukey multiple comparison test). (b) NSC-34/NF-κB luc cells were pretreated with 1 μM WFA for 20 min. After the treatment, cells were exposed to TNF-α (40 ng/ml) (*n* = 4, one-way ANOVA; Tukey’s multiple comparison test). (c) Cell death assay performed with BV2 cell by addition of lipopolysaccharide (LPS) and withaferin-A (WFA) in dimethyl sulfoxide (DMSO) (one-way ANOVA; Bonferroni’s multiple comparison test; *n* = 6). (d) Cell death difference observed in NSC-34 cells by addition of TNF-α and withaferin-A (WFA) dimethyl sulfoxide (DMSO) (*n* = 4; one-way ANOVA; Bonferroni’s multiple comparison test). *P* < 0.0001 ***)

To assess the effect of WFA treatment on NF-κB activity of microglial cells and neurons, we used BV2/NF-κB-Luc and NSC-34/NF-κB-Luc cells. We pretreated BV2/NF-κB-Luc cells with LPS for 2 h and then exposed the cells to 2.5 μM of WFA for the next 2 h in LPS-containing media. LPS treatment alone has induced luciferase activity by 4-fold in comparison to the control DMSO-treated group (Fig. 1a). Further, treatment with 2.5 μM WFA significantly reduced luciferase activity in comparison to the LPS treated group. We also checked the effect of pretreatment of WFA on NF-ĸB activity in the neuronal cell population. We pretreated NSC-34 /NF-κB-Luc cells were with 1 μM WFA for 20 min and after the cells were exposed to TNF-α (40 ng/ml) for 4 h (Fig. 1b). We observed reduced NF-κB luciferase activity in the WFA-pretreated group in comparison to the TNF-α-treated group alone. We did not observe any cell death in any of the treatment groups (Fig. 1c, d).

### WFA Treatment Reduces TDP-43 Inclusions and Improves Cognitive Function in TDP-43^G348C^ Mice

**Fig. 2:**
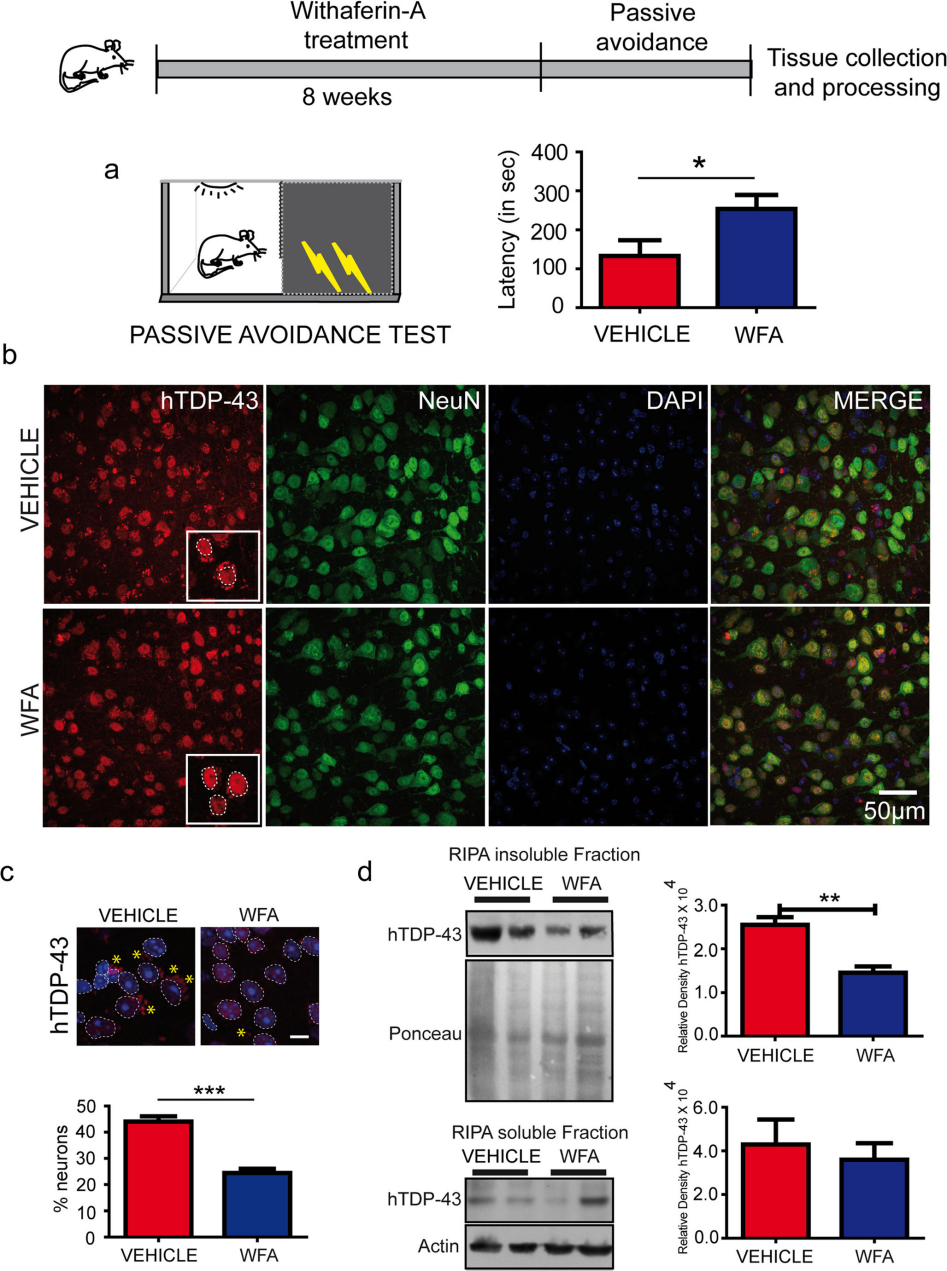
WFA treatment reduces TDP-43 inclusions and improves cognitive function in TDP-43^G348C^ mice. (a) Passive avoidance test performed after 8 weeks of saline and withaferin-A treatment in hTDP-43^G348C^ mice. Graphs showing freezing time in second spent by mice (*n* = 7; Unpaired t-test * *p* < 0.05). (b) Representative pictures showing TDP-43 (red), NeuN (green), and DAPI (blue) in the brain sections of vehicle or WFA-treated mutant hTDP-43^G348C^ mice. (c) Data showing stereology of % neurons showing TDP-43 cytoplasmic aggregation in vehicle or WFA-treated mutant hTDP-43 mice. Five to six cortical sections from each mouse were analyzed. Further, to make a comparison and perform statistical analysis, 3 mice were used from each group. (d) Representative immunoblots and quantification of RIPA-insoluble and RIPA-soluble fractions of brain from *n* = 3 to 4 independent mice from both hTDP-43^G348C^ mice. RIPA-insoluble TDP-43 or RIPA-soluble TDP-43 was normalized with ponceau or actin, respectively. Statistical analysis used was unpaired *t* test. Data represents mean ± sem. ** *p* < 0.01

A dose of 5 mg/kg of body weight of (WFA) was administered intraperitoneally once every 2 days for 8 weeks in 10-month-old hTDP-43^G348C^ mice. The control group has received an equal volume of saline. Randomized equal numbers of both males and females were used for the study. During the pathological stage, the hTDP-43 mutant mice exhibit cognitive defects [21]. To test the therapeutic potential of WFA on memory function, we used a passive avoidance test. In this memory task, WFA-treated mice exhibited better performance than the saline-treated group. Figure 2a shows the results of performance of all mice during the passive avoidance test. The average latency of mice to enter the dark chamber was significantly higher in the WFA-treated TDP-43^G348C^ mice than vehicle-treated TDP-43^G348C^ mice.

Further, to understand the impact of WFA treatment on TDP-43 pathology, we examined the levels of RIPA-insoluble and RIPA- soluble human TDP-43 in the brain of these mutant mice. Western blot analysis revealed a significant reduction of brain insoluble hTDP-43 protein upon WFA treatment in comparison to vehicle treatment. On the other hand, WFA treatment did not affect the levels of RIPA-soluble human TDP-43 in the brain (Fig. 2c, d). Further immunohistochemistry revealed that NeuN+ cells in the brain of WFA-treated mice had less TDP-43 aggregates than saline-treated mice (Fig. 2b, c). These results indicate that WFA treatment reduced the aggregation of TDP-43 in the brain of TDP-43^G348C^ mice.

### WFA Reduced NF-κB Activation and Microglial and Astrocyte Activation in the Brain of TDP-43^G348C^ Mice

**Fig. 3:**
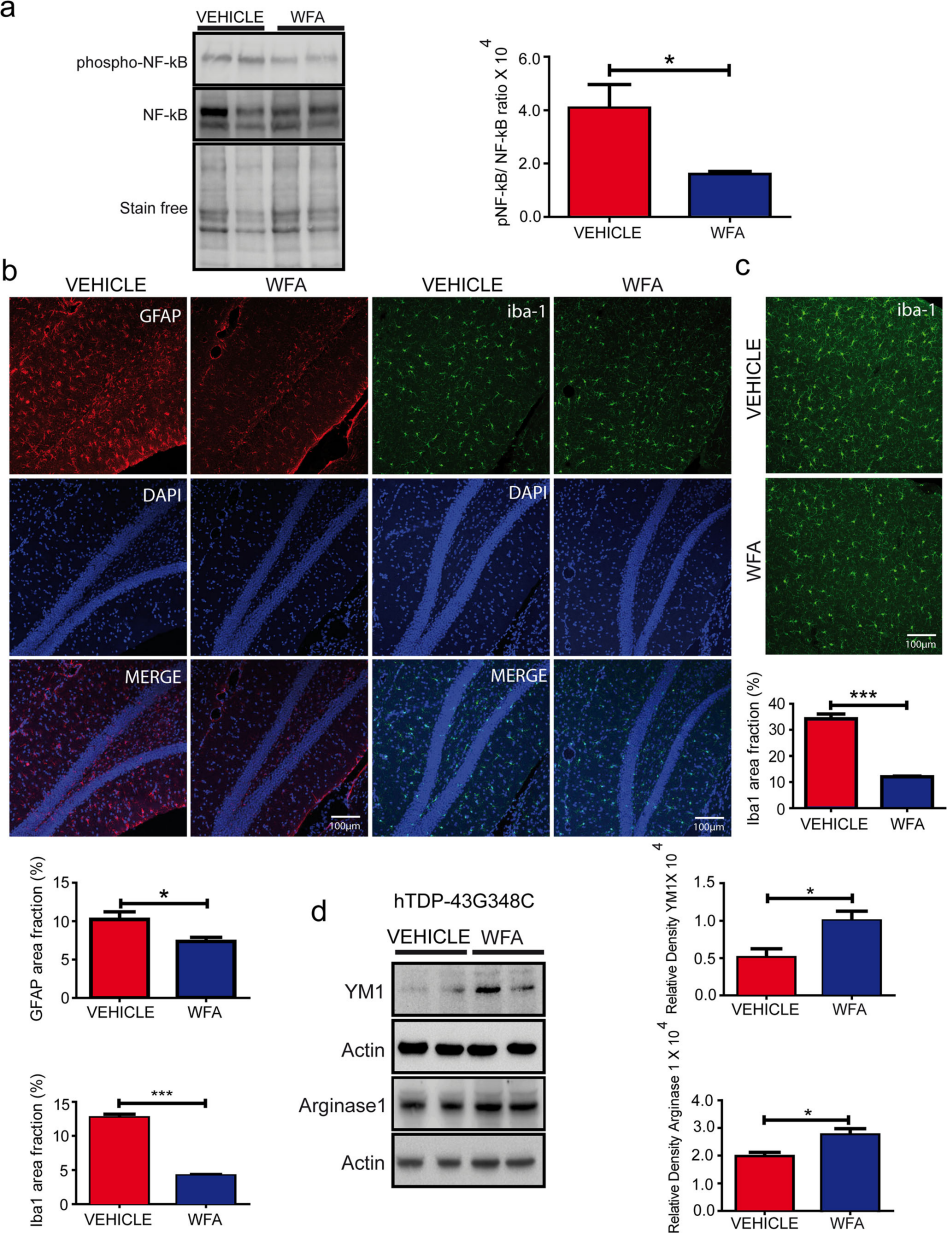
WFA reduced microglial and astrocyte activation in the brain of TDP-43^G348C^ mice. (a) Representative immunoblots and quantification of NF-ĸB and phospho-NF-ĸB levels in the brain from *n* = 4 independent mice from both saline and WFA-treated hTDP-43G348C mice. (b) Representative immunostaining showing astrocytes in the hippocampal region of 1-year-old TDP-43^G348C^ mouse brains treated with vehicle or WFA. The graph represents quantification of % area covered by the GFAP+ cells, using multiple sections from *n* = 3 mice, unpaired *t* test; * *p* < 0.05. Representative immunostaining of Iba1+ staining in the (b) hippocampus and in the (c) cortex of vehicle or WFA-treated mice (*n* = 3). The graph represents the % area fraction, unpaired *t* test; *** *p* < 0.001. (d) Representative immunoblots and quantification of YM1 and arginase1 levels in the brain from *n* = 4 independent mice from both saline and WFA-treated hTDP-43^G348C^ mice

During aging, there is occurrence of neuroinflammation in the brain of transgenic mice expressing mutant hTDP-43 proteins [21]. To study the effects of WFA on neuroinflammation, we checked the phospho-NF-kB levels, a pro-inflammatory marker. Our results have revealed that WFA treatment significantly reduced the phospho-NF-ĸB protein levels in the brain of TDP-43^G348C^ mice without changing the total NF-ĸB protein levels (Fig. 3a).

Further to study the glial inflammatory state, we used immunostaining for GFAP and iba1, markers of astrocytosis and microgliosis, respectively. The percent area analysis in the brain sections revealed that WFA treatment of TDP-43^G348C^ mice has significantly reduced the area fraction occupied by Iba1^+^ cells by about 3-fold in both the cortex and the hippocampus (Fig. 3b, c). Moreover, in the vehicle-treated group, the Iba1+ cells had larger cell soma while the processes were thick and short in comparison to the WFA-treated group. Microglia have been found to regulate either a neuroprotective state or neurotoxic state by class switching between M2 and M1 phenotypes, respectively [23]. To study the impact of WFA on microglial phenotypes, we checked M2 phenotype markers, YM1, and arginase 1 in the brain of TDP-43^G348C^ mice. Interestingly, the immunoblot of the brain lysates revealed that WFA treatment significantly increased the levels of both YM1 and arginase 1 in this mouse model of FTD/ALS (Fig. 3d).

Further, the area fraction analysis revealed a significant reduction of about 50% in the immunoreactivity of GFAP protein in the WFA-treated group compared to the saline-treated group in the hippocampus of TDP-43^G348C^ mice. Further, immunohistochemistry has revealed a similar pattern of staining as seen in microglia. The GFAP immunoreactivity had revealed thick processes and large cell soma size of astrocytes in the vehicle-treated mice brain. In comparison, the WFA-treated mice exhibited brain astrocytes with small cell soma and thin processes (Fig. 3b). In total, the data suggest that the WFA treatment has reduced the neuroinflammation and promoted the microglial neuroprotective M2 phenotype in this mouse model of FTLD.

### WFA-Activated Autophagic Pathways

**Fig. 4:**
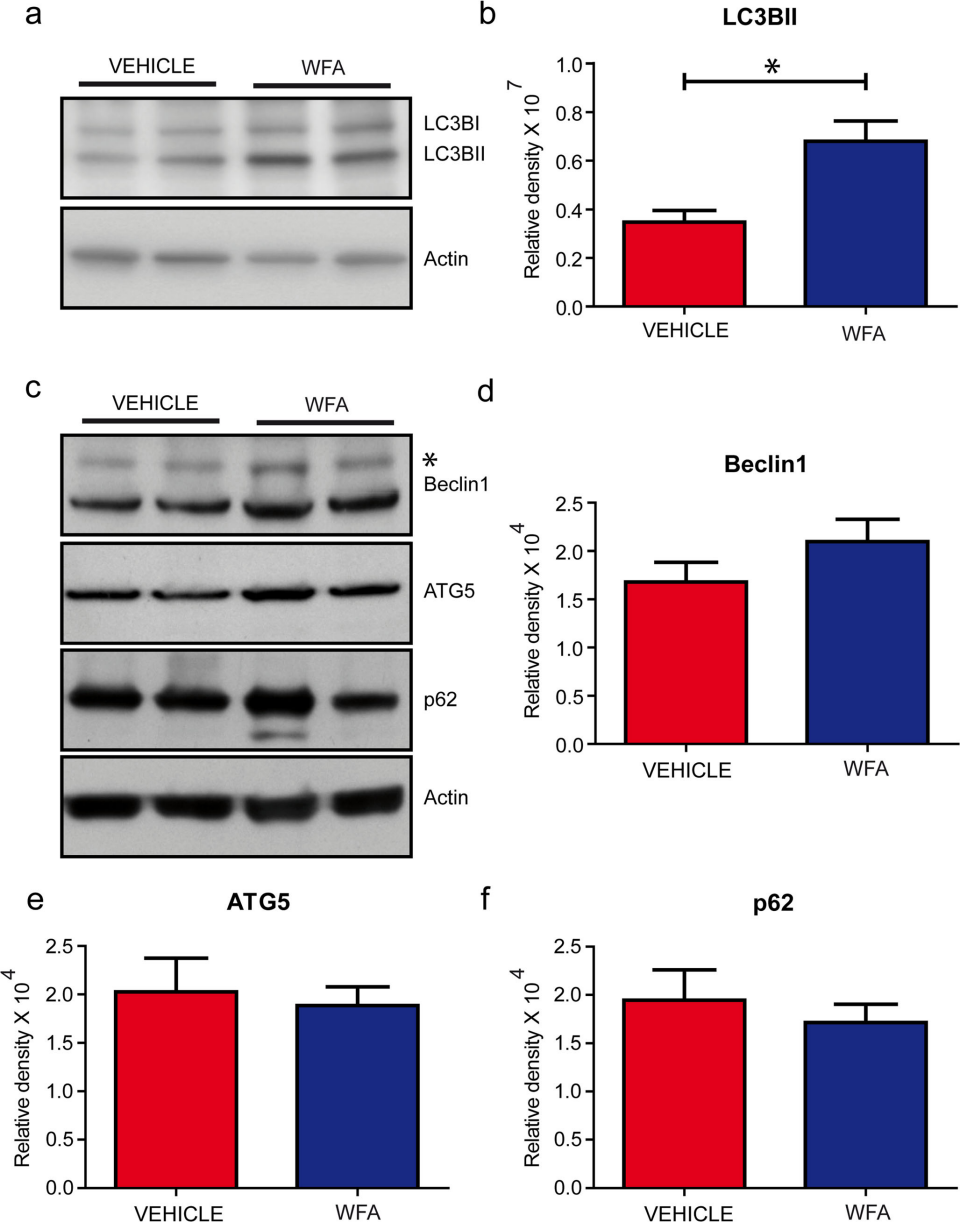
WFA activated autophagic pathways. (a) Representative immunoblots showing autophagic markers LC3BII and (b) quantitative data of LC3BII blot in the brain of hTDP-43^G348C^ mice received vehicle or WFA treatment. Representative immunoblot of (c) autophagic markers and data showing normalized density of (d) Beclin-1, (e) ATG 5, and (f) p62 in TDP-43^G348C^ mice that received either saline or WFA (*n* = 3 to 4). Protein levels were normalized using actin a Statistical analysis used was unpaired *t* test. Data represents mean ± sem. * *p* < 0.05

Previous studies have shown NF-κB as a negative regulator of autophagic pathways [24] and that WFA acted as an inducer of autophagy in hepatocellular carcinoma [25]. Here, we have determined by immunoblotting of brain extracts the levels of LC3B-II, a marker of autophagosome formation [14]. The levels of LC3B-II were significantly increased by twofold in TDP-43^G348C^ mice treated with WFA when compared to the vehicle-treated group (Fig.4a, b). The levels of other autophagic markers including Beclin-1, p62, and ATG-5 remained unchanged (Fig. 4c–f). From these results, we conclude that WFA acted as an inhibitor of NF-κB signaling as well as an inducer of autophagy in the brain of TDP-43^G348C^ mice.

## Discussion

The current study shows that WFA treatment reduced TDP-43 aggregates and NF-κB-dependent inflammation whereas it increased levels of LC3BIIin the brain of TDP-43^G348C^ mice. TDP-43 proteinopathy is a pathological hallmark of many neurodegenerative diseases including FTLD, ALS, Alzheimer’s disease, and LATE [2, 4, 10]. Previously, it was reported that expression of hTDP-43 mutants in mice induced during aging NF-κB activity in the brain [15]. Such NF-κB activation has also been seen in the postmortem spinal cord samples from ALS cases [15]. NF-κB is a transcription factor that regulates inflammatory function including genes encoding cytokines and chemokines [15, 17]. Different studies have shown that suppressing the NF-κB pathway in different neurodegenerative diseases including ALS mitigated pathological phenotypes [15, 17, 26, 27]. Our group recently reported that neuron-specific expression of the super-repressor form of the NF-κB inhibitor reduced inflammation and mitigated ALS/FTLD-like pathological changes in mouse models of ALS and FTLD [9]. Microglia-specific NF-κB inhibition also reduced inflammation and extended the survival of SOD1^G93A^ mice [17]. Moreover, AAV-mediated delivery of an antibody targeting the interaction of TDP-43 with p65 NF-κB reduced NF-κB activation and TDP-43 pathology in mouse models of ALS/FTD [10].

The present study shows that WFA reduced the NF-κB activation induced by LPS or TNF-α in cultured microglia and neuronal cells (Fig. 1a, b). In addition, WFA treatment attenuated NF-κB activation, microgliosis, and astrocytosis in the brain of TDP-43^G348C^ mice (Fig. 3). Neuroinflammation including astrocyte and microglia activation has been found neurotoxic in various neurodegenerative diseases [21, 28]. Different studies have also reported that neurotrophic factor release from glial cells has been found to be neuroprotective [16, 29]. Hence, glial response plays an important role in disease progression in different neurodegenerative diseases. Microglia has been found to regulate neuroprotective state or neurotoxic state by class switching between M2 and M1 phenotypes, respectively [23]. Our previous study has also shown that a root extract of *Withania somnifera* has also been found to reduce neurotoxicity and induce M2 phenotype of microglia [16]. Since we found WFA treatment reduced iba1 immunoreactivity in the brain of TDP-43^G348C^ mice, we further studied M2 markers in the brain. Immunoblot of brain extract has shown increased YM1 and arginase1 following WFA treatment which reflects M2 neuroprotective phenotypes of microglia. WFA treatment was also found to reduce neuroinflammation, neuronal apoptosis, and promote functional recovery also in traumatic brain injury and spinal cord injury [30, 31]. Previously, our group has also reported that WFA reduces inflammation and ameliorates ALS-associated motor function in SOD1^G93A^ mice [19]. There are reports that WFA directly binds and inhibits the IKKβ kinase activity causing inhibition of NF-κB signaling [32, 33]. However, WFA is a poor IKKβ inhibitor and its inhibition of NF-κB signaling is better explained by its covalent binding to cysteine-397 of the NF-κB essential modulator (NEMO) causing a disruption of complex reorganization into ubiquitin-based signaling structures which leads to reduce IKKβ and NF-κB activation [34].

Autophagy is an intracellular process that allows recycling of cytoplasmic constituents in order to maintain cellular homeostasis. In addition, autophagy also participates in degradation of the intracellular aggregated proteins [13, 27]. Studies have shown that induction of autophagy reduces the levels of different aggregated protein in neurodegenerative diseases including Alzheimer’s disease (AD), ALS, and Huntington’s disease [35–37]. It has been reported that inhibiting NF-κB signaling pathway leads to the activation of autophagic pathways including increasing the levels of cellular LC3BII [9, 24, 38]. Our results indicate that in addition to inhibition of NF-ĸB, WFA treatment also induced the levels of LC3BII in the brain of TDP-43^G348C^ mice. LC3BII upregulation is known to be a reliable indicator of the autophagic process which confirms the fusion of the autophagosome with the lysosome. However, it is noteworthy that WFA did not induce expression of other autophagy markers like Beclin-1 and Atg-5 in TDP-43^G348C^ mice (Fig. 4). It is unclear why WFA treatment, an inhibitor of NF-κB signaling, led to such differential expression of autophagy-related proteins whereas selective neuronal expression of a IκB-super-repressor transgene in TDP-43^G348C^ mice led to co-induction of LC3BII, Atg-5, and Beclin-1 expression [9].

Our study revealed that WFA treatment reduced the levels of RIPA-insoluble TDP-43 in the brain of TDP-43^G348C^ mice by approximately 50% (Fig. 2). In addition, WFA reduced the percentage of cortical neurons showing TDP-43 cytoplasmic mislocalization. WFA acted as an autophagy inducer (Fig. 4) which can explain in part the clearance of cytoplasmic TDP-43 aggregates in the brain of WFA-treated mice. The mitigation of TDP-43 pathology by WFA treatment was associated with amelioration of cognitive performance.

In conclusion, these results suggest that NF-κB signaling and autophagy pathways constitute therapeutic targets for TDP-43 proteinopathy in neurodegenerative diseases. This study suggests the therapeutic potential of WFA for the treatment of neurodegenerative diseases with TDP-43 protein aggregation. A recent pharmacokinetic study with advanced-stage high-grade osteosarcoma patients has shown that oral WFA was well tolerated without toxicity at a dose of 216 mg per day [39]. Nonetheless, further studies may be required to assess the safety of long-term treatment effects of WFA before considering this compound in clinical studies for neurodegenerative diseases.

## Electronic Supplementary Material

ESM 1 (PDF 498 kb)
